# Floating nationality: its paradoxes in theory and practice

**DOI:** 10.3389/fsoc.2026.1830290

**Published:** 2026-07-02

**Authors:** Johannes Kögel

**Affiliations:** Institute of Social Medicine and Health Systems Research, Otto von Guericke University Magdeburg, Magdeburg, Germany

**Keywords:** belonging, differentiation, floating signifier, identity, nationality, paradox, postcolonial, suture

## Abstract

Nationality can be understood not simply as legal membership in a political community but as a complex and unstable means of signifying collective identity. References to nationality do not carry a fixed meaning; instead, they function as signifiers that attempt to create coherence and continuity in how groups understand themselves. Nationality operates at the intersection of identity (what a group believes it is) and membership (who belongs), yet emphasizing one dimension inevitably obscures the other. As a result, nationality often appears stable while remaining internally ambiguous and context-dependent. It acts as a “floating signifier” whose meaning shifts across political, cultural, and social settings. Nationality, in its ambiguous two-dimensionality as membership and identity, or form and content, produces paradoxical experiences in lived reality, even more so in postcolonial contexts. While the notion of nationality contains inherent paradoxes, the postcolonial context—through its engagement with colonial structures and legacies, and its efforts to negotiate identity and belonging—reveals these ambivalences, contradictions, and discrepancies in particularly pronounced ways.

## 1 Introduction

Recent social science research on nations and nationality increasingly treats national identity as a multidimensional, dynamic social construct shaped by psychology, institutions, and digital communication. Scholars emphasize that national identity consists of different forms of attachment, such as nationalism, political patriotism, and cultural patriotism, which influence political attitudes, including views on immigration and social cohesion. These different dimensions of national identity can shape attitudes toward migrants and broader political preferences in distinct ways (Indelicato and Martín, 2024; Lindstam et al., 2021). Contemporary studies also explore the cognitive and emotional foundations of nationalism, arguing that collective memory, imagination, and emotions such as pride or fear allow individuals to internalize the nation as a shared identity, linking social psychology and neuroscience with nationalism studies (O’Mara, 2026). Another emerging direction is the study of “digital nationalism,” which examines how social media, online discourse, and technological competition produce new forms of national identity and mobilization in everyday communication (Ahmad, 2022; Li et al., 2026). Overall, research corroborates the widely held notion of nationality as an evolving social phenomenon (rather than a fixed cultural trait) that is continuously reproduced through political narratives, institutions, and digital media.

In addition, nationality is widely held to be a particular source of identity, much like class or gender, which are also responsible for forming specific identities. The particularity of nationality in this regard lies in its usurpation by the state.^1^ By denoting belonging to, or membership in, a nation-state, nationality signifies not only national identity but also citizenship. Rather than giving this notion a singular or new name (such as “nation-statility”), the term “nationality” was adopted (for understandable strategic reasons)^2^ and endowed with an official, formal, and politically effective frame. The term’s ambiguity is already present in the notion of the “nation,” where it often stands for the “nation-state.” In this case, the nation-state always functions as a legitimate specification—unlike nationality (or “nation-statility”).

This difference—or, more aptly, what may be called différance (Derrida, 1982), due to the simultaneous differentiation and mutual reference of both connotations of the term—allows for its strategic utilization and co-optation. One example, to which I will return below, is the positive description and valorization of the “Zimbabwean” by Zimbabwean nationals in South Africa in order to grant themselves an authentic speaking position in political discourse and to make legitimate claims—a discourse accessed through one’s citizenship. Such dynamics may occur more frequently in younger nation-states and thus often in postcolonial contexts. However, the same rationale is utilized by national(ist) movements aiming for separation and national sovereignty across the globe.

This paper is dedicated to analyzing the notion of nationality in its components and to apply to the postcolonial context. Nationality will be broken down into its inherent differentiation, its functioning as a floating signifier, and the suturing effect that binds national categorization to subjective identification in terms of nationality. As both a concept and a lived reality, nationality comprises two dimensions: national identity and belonging or membership—more precisely, form and content—each of which exists in its own right while remaining interdependent with the other.^3^ The paradoxes implicit in nationality, with particular emphasis on its salience in postcolonial contexts, will be examined alongside empirical cases.

## 2 Methods and material

This paper applies a sociological lens to the question of the nation and thereby focuses in particular on nationality. In doing so, it is at once both more basic and derivative. Nationality can be traced in its concrete use in social discourse and practice, for example in ascriptions and descriptions made by oneself and by others. However, being derivative does not mean that the entity of the nation necessarily precedes nationality. There are good reasons to argue that there is no nation without people ascribing nationality to others and to themselves. Indeed, claims to and ascriptions of nationality may precede the nation itself, or may even amount to national enunciations. This perspective is suggested when nations are understood as “imagined communities” (Anderson, 2006).^4^

Nationality is further complicated by the fact that it can refer to different types of nations, such as those typified by Hadžidedić (2025). These may, as mentioned before, be tied to statehood, though not in all cases (for example, stateless nations). At the same time, nationality may denote formal membership in a political community, that is, citizenship.

The examination of nationality in this paper is theoretical in nature. The theoretization of nationality as a floating signifier is grounded in a conceptual analysis (Kögel, 2025) based on an empirical study (Kögel, 2024). While the empirical study has been conducted in a postcolonial context, the conceptualization of nationality offered claims general applicability. This conceptualization will be applied *expressis verbis* to the postcolonial context, since—as I will argue—it exemplifies the notion of nationality in a particularly pronounced way. The conceptual paradoxes discussed as well as the empirical cases outlined are drawn from the existing literature on nationality.

## 3 Nationality: its form and content

Nationality, I will argue, can be defined through a dual structure of form and content. Its content concerns the contingent characteristics of particular nationalities—however arbitrary, variable, and historically shifting they may be—which are performative in nature. They must be enacted in order to exist; that is, they must be taken up and given life by subjects, constituting nationality as a form of identity. As such, nationality can only exist in the plural: each nationality emerges in differentiation from others, while nationality as a general concept provides the overarching frame or scaffolding—a floating signifier that can be filled with particular (discursive) content, which must be sutured to subjects in order to materialize.

Its form consists of a distributive logic. Given the mutually exclusive system of differentiation among nationalities, it operates like a classificatory system that organizes belonging, subsuming subjects under respective nationalities and thereby designating their membership, or in the case of nation-states, their citizenship.

### 3.1 National identity

#### 3.1.1 Differentiation

According to Ferninand de Saussure, all signifiers gain their identity only by differentiation to other signifiers, hence being a production of a network of differences. The same, according to Jacques Derrida (1982), applies not only to language, but to reality, at least our perception of it as well as our thoughts and ideas, all gaining content solely in relation, i.e., differentiation, to others. Hereby, Derrida regards the process of differentiation along the dimensions of space and time. No presence is stable or has a substance, or a self-identity. Hence, no identity can make a firm case for what it is other than hinting, deferring to its traces, its absences, its relationality to others.

If this applies to the identity of every denomination it certainly also applies to what we refer to as social identities (such as nationalities). Here, we also have the advantage of having empirical cases at our disposal. Drawing on experiments from social psychology, we may think of the Robbers Cave Experiment (Sherif et al., 1961; Fine, 2004), where two camps of boy scouts started to label themselves as soon as they learnt of the existence of the other camps and both took over mutually opposite ascriptions and stereotyping of themselves and the others—and this only within the short period of four days. Social Identity Theory (and Social Categorization Theory) proclaim that simply grouping random groups together, for example by giving them a team name, no matter their internal heterogeneity or inter-group homogeneity with other teams, suffices to create insider and outsider dynamics (Tajfel and Turner, 1979; Turner et al., 1987). Hereby, the chronological order is decisive. In the words of Kwame Anthony Appiah (2018, p.30): “Labels came first, then, but essences followed fast.”

This very same mechanism occurs in the process called schismogenesis. While the rationale of opposing group characteristics is the same, schismogenesis highlights the process that can be observed of groups that foster and increase their mutually exclusive descriptions of selves and the others, furthering the perceived divide between them (usually somehow neighbouring groups as some knowledge about each other is necessary). Gregory Bateson (1936) grounded this insight on his research of the Iatmul. David Graeber and David Wengrow (2021), scanning the total of human history, emphasize a few examples, such as the ones between Athens and Sparta, highland Teotihuacan and lowland Maya, forager cultures of California and the American Northwest Coast, or the Wendat and French settlers in North America in the 17th century. They however stipulate to see the same principle to apply also among the modern nation-states: “If “national character” can really be said to exist, it can only be as a result of such schismogenic processes: English people trying to become as little as possible like French, French people as little like Germans, and so on” (Graeber and Wengrow, 2021, p.58).

#### 3.1.2 Floating nationality

According to this mutual differentiation, we must comprehend identity as formed through opposition. As a consequence, the task at hand according to Ernesto Laclau (2005, p.69) is as follows: “Given that we are dealing with purely differential identities, we have, in some way, to determine the whole within which those identities, as different, are constituted.” Laclau describes this whole as an “impossible object”: a necessary yet “failed totality” that nonetheless guarantees signification and identity, made possible when a particularity claims universal meaning (Laclau, 1995, 2005).

If nationality were defined solely through differentiation, national signifiers would be empty. In practice, however, nationalities are filled with stereotypical and self-ascribed attributes shaped by identity politics. Through mutual differentiation, these attributes become relational and oppositional, varying by context. National identity is thus context-dependent and unstable, turning nationalities into floating signifiers—terms without fixed meaning that absorb meanings imposed upon them.

Claude Lévi-Strauss (1987) introduced the notion of the floating signifier as a term devoid of determinate meaning, capable of absorbing any meaning—a “zero symbolic value.” Such signifiers guarantee the functioning of symbolic systems by stabilizing otherwise sliding relations between signifier and signified. Applied to nationality, this highlights a tension: while nationality is often defined as membership in a nation-state, membership in a “nation” itself remains conceptually open and contested.

Nationality also functions as a stabilizing signifier that halts the endless sliding of national differences. As long as nationality signifies state membership, the absence of essential characteristics poses no problem. Thomás de Zicman Barros (2023) likens this to Wittgenstein’s propositions that are “exempt from doubt”: nationality is typically taken for granted, as reflected in migrants’ narratives (Kögel, 2024).

Historically, nationality carried a democratic promise of equality, dignity, and self-determination, especially in anti-colonial struggles. Today, nationality largely signifies legal membership in a nation-state. Yet nationality functions not only as a legal status but as a strategic and affirmative identity resource. Even as a derogatory ascription, it remains a discursive position that migrants reclaim and re-signify positively (Kögel, 2024).

To be successful, nationality as both identity and belonging must surely be imbued with life and is therefore invested with national symbols (Billig, 1995), invented traditions (Hobsbawm and Ranger, 1984), and emotions (Feinstein, 2024). However, in its dual structure—as identity and belonging—nationality constitutes this process (while at the same time depends on its performativity) by means of operating as a floating signifier.

Nationality does not derive meaning from shared substance but from classification and exclusion—it belongs here because it is not elsewhere. This formal logic already unites nationality as identity (difference) and nationality as belonging.

#### 3.1.3 Suture

Stuart Hall (1996b, pp.5-6) conceptualizes identity through the notion of *suture*, the temporary attachment between discursive positions and the subjects capable of occupying those positions:

*I use “identity” to refer to the meeting point, the point of suture, between on the one hand the discourses and practices which attempt to “interpellate,” speak to us to hail us into place as the social subjects of particular discourses, and on the other hand, the processes which produce subjectivities, which construct us as subjects which can be “spoken.” Identities are thus points of temporary attachment to the subject positions which discursive practices construct for us.*

Identities are thus not fixed essences but contingent points of attachment. As a concept rooted in theories of the signifier, suture stands in close relation to the floating signifier.

This can be compared to Jacques Lacan’s “quilting point,” described by Slavoj Žižek (2012) as the moment where the signifier “falls into the signified.” In systems of purely differential identities—as described above—, meaning requires the patching up between signifiers and signified:^5^

*“if the identity of a signifier is nothing but the series of its constitutive differences, then every signifying series has to be supplemented—“sutured”—by a reflexive signifier which has no determinate meaning (signified), since it stands only for the presence of meaning as such (as opposed to its absence)”* (Žižek, 2012, p.584).

Jacques-Alain Miller (2012) introduced “suture” to account for the subject’s place within structure. Using the metaphor of zero, he showed how an absence can nonetheless be counted: zero, operating as a “stand-in,” names nothing, thereby making it symbolically operable. The subject, similarly, is the countable name for a lack—an excess that allows structure to function. This subject is not a conscious agent but an effect of the symbolic or discursive order.

Hall (1996b) adapts this structural notion of suture to address identity. For him, suturing is not one-sided: subjects must invest in the positions offered to them. Identity, or identification, names this articulation between discursive positioning and subjective uptake.

Although Lacanian theorists would resist Hall’s emphasis on agency, both perspectives agree that subjects are produced within discourse (or the symbolic order). In this sense, speaking as “I/We” constitutes the subject position. Identity here is not essential but “strategic and positional” (Hall, 1996b, p.2).

Nationality as a floating signifier enables this suture. Any particular national identity does not possess fixed content; it’s meaning shifts across contexts and contrasts with other nationalities. What stabilizes it is not essence but the form of belonging it signifies. Nationality provides an anchoring framework that links identity claims to questions of rights, belonging, and legitimacy. “Nationality” basically allocates meaning without supplying substance. There is no essential national identity behind its expressions; identity exists in performative enactment. As Butler (2004) argues for gender, nationality is constituted through its expressions rather than preceding them.

Accordingly, there are no essential national identities, only situational, relational articulations shaped by context and opposition.^6^

### 3.2 Nationality and citizenship

So far, we have discussed nationality as national identity, predicated on its characteristics of mutual differentiation and the suturing of floating signifiers.

In the global political system, the world consists of mutually exclusive (or sovereign) nation-states, thereby ascribing to each holder of a nationality at least one decisive official national belonging, that is, citizenship. Consequently, the two often have conflated meanings, as also noted by Erin Chung (2017, p. 432), who states that “the terms, “nationality” and “national”, are used to refer exclusively to legal juridical membership in a nation-state.” Although these terms refer to the same basic phenomenon, different connotations can nevertheless be distinguished. Nationality may be understood as emphasizing belonging and affiliation—often in an emotional sense—and may serve as a source of identification, whereas citizenship stresses formal or legal membership in a polity and emphasizes the rights (and duties) associated with that status.

For example, Hall (1992, p. 292) argues that “a nation is not only a political entity but something which produces meanings—a system of cultural representation. People are not only legal citizens of a nation; they participate in the idea of the nation as represented in its national culture.” Saskia Sassen (2002, p. 278) recognizes a distinction between the two terms within their legal dimensions:

*Today the terms citizenship and nationality both refer to the national state. In a technical legal sense, while essentially the same concept, each term reflects a different legal framework. Both identify the legal status of an individual in terms of state membership. But citizenship is largely confined to the national dimension, while nationality refers to the international legal dimension in the context of an interstate system.*

Lived experience reveals various forms of how citizenship is enacted, which are described with terms such as “transnational citizenship” (Fox, 2005), “symbolic citizenship” (Werbner, 2000), and “diasporic citizenship” (Werbner, 2002). Postcolonial citizenship—or citizenship in the Global South—often exhibits particular characteristics, such as a certain flexibility (Ong, 1998; Nyamnjoh, 2007) or graduality (Neocosmos, 2010) (see also Kögel, 2024, pp. 143–145). As such, the lived form of citizenship shapes understandings and performances of nationality, as will be shown below. For this argument, it suffices to note that nationality has a dimension of membership or formal belonging, which—within the system of sovereign nation-states—takes the form of citizenship.

Thus, while national identity may be discursively produced, it nevertheless shapes one’s social habitus (Wodak et al., 2009) and its citizenship dimension has tangible consequences: it determines where one can travel, where one holds rights and duties, and what opportunities are available. Nationality, as described above—being predicated both on structural conditions and on individuals’ participation—certainly possesses a discursive dimension, but in practice it functions as a solid mediator of social and political life.

### 3.3 Form and content

Consequently, I will differentiate between the form and content of nationality. While the latter refers to what nationality is made of—often labelled national identity—the former denotes its dimension of belonging or membership.

Unlike conceptualization of the “nation form” (Balibar, 1990; Goswami, 2002; Mutman, 2008), I will regard the form of nationality as its formal component: the belonging that is classified and distributed according to mutually exclusive categories. The fact that individuals may possess double or multiple nationalities, that is, citizenships, does not contradict this principle, since having several nationalities does not merge them into a new one in terms of citizenship. In terms of national identity, however, this may give rise to ambivalent or novel experiences and perceptions of national belonging, thereby underlining the relative independence of these two dimensions.

In this sense, the two dimensions may even be largely indifferent to one another. Where the formal aspect is decisive—for example, when determining which citizenship one holds while travelling to another country or seeking employment abroad—one’s national identity is generally irrelevant. Conversely, citizenship alone may not suffice for some observers to recognize a person’s legitimate or authentic claim to belonging to a national group if that person is perceived to lack certain markers associated with such belonging, for example in terms of accent, appearance, behaviour, or affiliations.

Of course, one could simply argue for the distinct use of national identity and citizenship as separate analytical categories—and indeed I endorse the argument advanced by Brubaker and Cooper (2000) not to use “identity” as a category of analysis. The difficulty, however, is that “nationality” is commonly used to encompass both dimensions in practice, both as a category of everyday practice and within academic discourse. I would also add that citizenship denotes the officially sanctioned belonging granted by the state and, as such, refers to specific rights and duties. These aspects are not necessarily implicit in nationality, where the emphasis lies rather on clear ascribability and distinct categorizability. For this reason, I will instead speak of the form and content of nationality.

The content refers to the “we” that is discursively constructed: the myths, traditions, narratives, and symbols involved, as well as the emotional and affective sense of belonging associated with them. The form, by contrast, consists of differentiation from “others,” the separation and distribution of individuals along distinct categories. Citizenship, in this sense, is simply the designation for the formal belonging to such a category.

## 4 Paradoxes involved in nationality

The mutual sliding of content and form highlights the paradox that each dimension loses significance when attention is focused on the other. For example, when formal belonging (such as citizenship) is at stake—e.g., in legal discourse or in instances of xenophobia where foreignness is reduced to possessing the correct identification documents—any markers of shared nationality, such as language, culture, ethnicity, or religion, become largely irrelevant. Conversely, when national identity is at stake, claims to formal belonging or membership are often disregarded—for instance, anti-migrant hostility frequently targets individuals who, although legal citizens, do not conform to certain stereotypical expectations. Accordingly, the common question, “Where do you come from?” directed at someone perceived as not fitting a national standard, is rarely resolved by citing formal membership or citizenship. Such answers often prompt further probing into where the person is “actually” from.

This paradox of shifting or mutually exclusive foci may, in fact, be necessary to navigate other paradoxes inherent in the concepts of the nation and the nation-state. These include the following:

### 4.1 Nation as an umbrella term

As John Joseph (2004) notes, the concept of a nation carries an inherent ambiguity or tension. On the one hand, it can refer to a people defined by common ancestry or nativity, as in the case of the Hebrew or Cherokee nations. On the other hand, it can denote the alignment of a sovereign government with a specific territory and its inhabitants, as exemplified by the British nation. A literal combination of these two meanings would result in a dystopian scenario in which a territory is inhabited exclusively by individuals born into it. Under the category of “nation,” Hadžidedić (2025) lists civic, post-colonial, post-imperial, ethno-linguistic, ethno-religious, complex, fractured, self-isolated, stateless, and internal nations, as well as nations-civilizations.

### 4.2 Nation versus nation-state

Particularly problematic are cases in which no congruence exists between nation and nation-state. Timothy Brennan (1990, p.45) accordingly points out: “As for the ‘nation’, it is both historically determined and general. As a term, it refers both to the modern nation-state and to something more ancient and nebulous—the “natio”—a local community, domicile, family, condition of belonging.”

At the same time, the nation-state claims sovereignty over a specific geographical territory and the population residing within its boundaries. Nations, however, often extend beyond territorial limits, crossing borders and forming diasporas (Appadurai, 1996). The increasing number of countries that grant expatriates the right to vote bears witness to the idea of national membership as transnational and diasporic.

What makes matters even more complicated is the confusing use of “nation” in place of “state,” which leads to conceptual misapplications, such as in “trans-” or “postnationalism,” as outlined by Sheila Croucher (2003).

### 4.3 Conflicting ideological roots

The nation-state as the natural embodiment of a people’s sovereignty is grounded in the Enlightenment ideal of autonomy. However, as Joseph (2004) points out: “For Enlightenment itself, to assert its sovereignty as the universal ideal, needs its Other; if it could ever actualise itself in the real world as the truly universal, it would in fact destroy itself.” As such, its promise is *per definitionem* impossible to fulfil completely.

The same intellectual roots also underlie the nation’s promise of liberation and self-determination for the people vis-à-vis the ruling class, as Eric Hobsbawm (1992, p. 20) explains:

*We cannot therefore read into the revolutionary ‘nation’ anything like the later nationalist programme of establishing nation-states for bodies defined in terms of the criteria so hotly debated by the nineteenth-century theorists, such as ethnicity, common language, religion, territory and common historical memories.*

Originally, then, the concept of the nation aimed to grant self-determination to the people and to secure independence from ruling elites. In practice, however, the opposite often occurred. The nation was appropriated by the state as a means of pacifying and controlling the broader population. The nation-state thus collapses the distinction between nationality and citizenship, positioning itself as both the source and guarantor of modern nationality.

### 4.4 Inner tensions

Nationality, as a signifier that seeks to stabilize identity and establish categories, assigns a seemingly fixed, essential meaning to groups of people—typically in relation to a nation-state. It can function to group individuals together while stereotyping or excluding others, or it can serve as a basis for positive self-identification among group members themselves. The central problem with the concept of nationality, however, is that it provides a framework for justifying both belonging and exclusion. Here, nationality as a vehicle for equal rights conflicts with nationality as a signifier of being an outsider (the aforementioned “other” nationality). With the nation-state as the custodian of the demarcation of nationalities, the nation-state prevents equal inclusion of individuals (Mamdani, 2020).

### 4.5 Inherent ambivalence of the nation

Homi Bhabha (1990) considers the nation to be essentially “Janus-faced,” containing two dimensions: the pedagogical and the performative. The former presents the nation as a historical continuity—timeless and given—and its people as a unified, homogeneous whole. The latter regards the nation as something that is constantly performed and reproduced through every day acts and speech, and therefore always changing and in the making. Nationality as national identity can thus be understood as something that occurs and is negotiated “in-between” (Bhabha, 1993). The constant re-articulation of national identity can therefore be seen as both reflecting the instability of nationality and contributing to the relative stability of its two dimensions.

Manu Goswami (2002) emphasizes the “doubled character of the nation-form” in being universal and particular at the same time. Nationality, as a universal form for collective identity, appears everywhere in a similar structural manner, while for each individual national identity it is crucial to be unique and sufficiently distinct from all others (see also Balibar, 1990).

## 5 Postcolonial nationalities

### 5.1 Postcolonial paradoxes

Shifting the focus to postcolonial nationality in particular, additional paradoxes become visible.

Colonial administrations initially aimed to reshape colonial populations into suitable members of the colonizing nation. When this governing approach, known as direct rule, proved ineffective, it was replaced by indirect rule, which followed the logic of “define and rule,” as described by Mahmood Mamdani (2012), deploying colonial inventions such as “ethnicities” (Ranger, 1996) or “tribes” (Comaroff, 1998). Under this system, native populations were organized into tribes, their differences were politicized, and people living in indigenous territories were treated as subjects rather than citizens. This legacy had profound consequences after independence. With the beginning of the postcolonial period, unresolved problems from earlier nation-building efforts resurfaced. As Mamdani argues, although indirect rule originally emerged as an alternative to nineteenth-century nation-building, it ultimately created the very conditions that shaped nation-building attempts in the twentieth century. By establishing separate political communities governed by different legal systems defined along ethnic lines, indirect rule institutionalized division. As a result, the postcolonial state became a contested space in which various groups struggled for political recognition and belonging.

After independence, many countries prioritized state-building—the creation of centralized governments—over nation-building, understood as the development of a shared national identity. This process was largely top-down and occurred without strong democratic institutions, which often contributed to the decline of multiparty democracy. As noted by Ali A. Mazrui (2001)—making the case for African states—many lost democratic governance within a decade of independence through military coups, authoritarian presidencies, civil wars, or the establishment of single-party systems.

Because national identity had not fully consolidated, attempts at democratization and multiparty politics often triggered conflicts over belonging and political rights. Questions emerged regarding who counted as a legitimate citizen, who could vote where, and who was eligible to run for office. This process—sometimes described as the “culturalization of citizenship” (Geschiere, 2011, p.339)—produced categories of intranational foreigners, in which certain groups were perceived as less legitimate or as “non-native” within the same country (Geschiere and Nyamnjoh, 2001; Geschiere, 2009).

Although anti-colonial nationalism initially united diverse ethnic and regional groups against colonial rule, the postcolonial period witnessed the re-emergence of ethnic and regional struggles over power, autonomy, and citizenship. Concepts such as autochthony—the claim to be native to a particular place—became new lines of political and social division (Geschiere, 2011). In many postcolonial countries, where colonial authorities had drawn arbitrary boundaries—territorial borders as well as classifications of races, tribes, and ethnicities—nation-building lagged behind state-building, as many administrative and political institutions (including boundaries, bureaucracies, and legal systems) were inherited from colonial rule. In other contexts, however, nation-building preceded state-building. As such, Partha Chatterjee (1993) argues that Indian nationalism developed a “cultural” or “spiritual” domain of the nation before political sovereignty, which later allowed the independent state to claim legitimacy as the expression of that nation.

Thus, while nationalism was directed against colonial rule, many administrative and political structures were retained, giving postcolonial states both an anti-colonial and a colonial character. This is why Frantz Fanon (1968) had an ambiguous stand towards nationalism: he views it as a necessary tool in the struggle against colonialism—in particular in form of creating a “national consciousness,” but warns that after independence it can become limiting or destructive unless it develops into a broader political and social consciousness.^7^

Furthermore, arbitrary borders that cut across populations with shared identities complicated efforts to construct cohesive national communities. In this sense, the “modern nation-state project,” based on the presumed congruence of nation and state reflected in a homogeneous people sharing a common language and culture, was “flawed from the outset” (Laakso and Olukoshi, 1996, p.11). Making the case for Africa, Mamdani (1992, p.2231) argues: “In Africa, more than in any part of the world, there is little coincidence between the history of nation formation and that of state formation, between social history and political history.”

Consequently, nationality was often set against other forms of identity and belonging—whether based on language, ethnicity, ancestry, religion, or culture. In this context, civic nationalism, which aspires to treat all citizens equally and assumes a state blind to difference, frequently conflicted with particularistic interests and efforts to recognize distinct group identities (Chatterjee, 2004).

Moreover, in many countries of the Global South, patterns of human mobility differ from those in the Global North. Migration is often less restrictive, with circular and cross-border migration playing a significant role (Bakewell et al., 2009), while informal labour markets are more prominent (Gagnon and Khoudour-Castéras, 2012).

Nation-building therefore had to bring together groups with different prior affiliations and forms of belonging, while also reconnecting populations that had been separated by colonial divide-and-rule or define-and-rule strategies. At the same time, newly independent states remained economically dependent on trade relations and political ties with former colonial powers, as well as on enduring global structures of inequality. Together with other inherited colonial legacies—epistemic, bureaucratic, and structural—these dynamics point to an ongoing “coloniality of power” (Quijano, 2000) or neo-colonial condition.

Also, while experiences of colonial domination are central to postcolonial national identities—often providing meaning through opposition to colonial rule—the essence of national identity is frequently sought in an imagined or reconstructed precolonial authenticity (Bhabha, 1990; Chatterjee, 1993).

Finally, as indicated above, citizenship takes different forms in the Global South. While citizenship in the Global North is associated with the provision of political, civic, and social rights, postcolonial citizenship does not necessarily entail social and economic rights in the same way (Sadiq, 2017). Moreover, whereas the acquisition of citizenship in the Global North generally follows the trajectory of formal naturalization, Kamal Sadiq (2009) demonstrates through his case studies how migrants can become “paper citizens” by circumventing this process. The various forms and degrees of postcolonial citizenship, particularly in terms of their inclusivity and capacity, inevitably shape understandings and perceptions of national identity and belonging.

### 5.2 Postcoloniality of nationality: the rhino in the room

However, I would not do justice to the understanding of postcoloniality by delimiting it to the territories that have experienced colonial rule in the past. As many compound terms made up by the prefix “post,” postcoloniality refers to the endurance of the colonial condition. Given the circumstance that modernity (and with it the modern nation-state) and coloniality were co-constituted, the postcolonial world did not enter Western countries by means of migration (according to the motto “We are here, because you were there!”), but rather is constitutive for a global interdependent transformation.

Mahmood Mamdani (2020, pp.1–2) states the following: “Modern colonialism and the modern state were born together with the creation of the nation state. Nationalism did not precede colonialism. Nor was colonialism the highest or the final stage in the making of a nation. The two were co-constituted.”

From this perspective, central features of the modern nation-state (e.g., systems of rule, categorizations of people, social engineering) first took shape in colonial settings before being adopted within European states themselves, a process Jordan Branch (2012) describes as “colonial reflection.” Similarly, Gurminder K. Bhambra (2014) highlights that in many European contexts the colonial state historically preceded the national state. In particular the Haitian Revolution denotes an event that shaped transformations in Europe, beginning with the French Revolution (James, 1963; Magubane, 2005).

This interpretation challenges the conventional narrative that traces the origin of the nation-state to the Peace of Westphalia in 1648, commonly regarded as the foundation of the modern system of sovereign states. Instead, Mamdani (2020), along with Aníbal Quijano (2000, p.558), identifies 1492 as a more meaningful starting point for the modern nation-state. In that year, the Castilian monarchy carried out the expulsion of Muslims (particularly Arabs and Berbers) and Jews from the Iberian Peninsula in an effort to construct a culturally homogeneous Christian polity. The completion of the “Reconquista” also coincided with the voyage of Christopher Columbus and the European “discovery” of the Americas. Overseas conquest was accompanied by comparable processes of violent exclusion and population removal.

The Reconquista represented an early attempt to purify a territory by removing those who did not conform to the dominant cultural and religious order. The principle underlying this effort—one territory, one religion, one empire—established a model that was later extended to colonial contexts. Those who refused conversion to Christianity were classified as uncivilized and thus denied full recognition as persons. The same logic was applied in the colonies: only those deemed “civilized” were to be accepted, while others were expected to undergo civilizing transformation or face expulsion or extermination.

Although the Treaty of Westphalia granted minority populations certain protections within Europe—aimed at preventing wars fought in the name of protecting co-religionists or ethnic kin—these principles did not extend beyond the continent. In colonial settings the opposite logic prevailed. European powers argued that “uncivilized” peoples lacked sovereignty, the very principle upon which the Westphalian order was based. This reasoning allowed colonial expansion to be framed not only as legitimate but even as a moral obligation.

The so-called civilizing mission in the colonies paralleled the nation-building processes occurring in Europe, which were largely imposed from above and drew inspiration from the Reconquista model. Colonial populations were expected to be reshaped into members of the colonizing nation.

**Figure 1:**
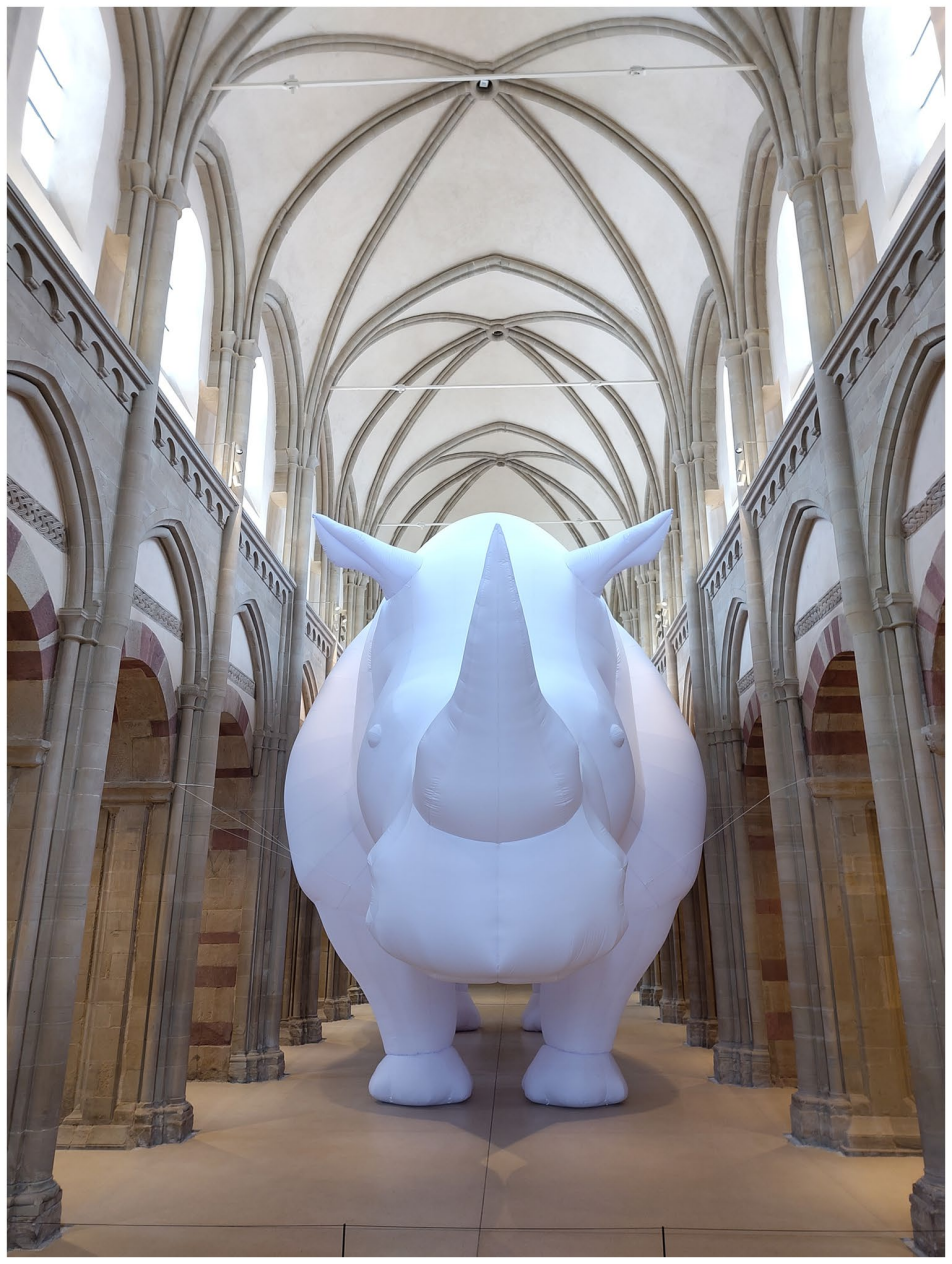
The rhinoceros in the room. Or: a tale of banality and evil by Itamar Gov at Kunstmuseum Magdeburg, 2026. Picture taken by the author. Printed with the kind permission of the artist.

This brief description should suffice to illustrate the co-constitution of the modern nation-state across the colonial divide, as well as to hint at the different trajectories it has taken depending on which side of that divide one considers. As a visual illustration of this co-constitution, I point to the art installation *The Rhinoceros in the Room. Or: A Tale of Banality and Evil* by Itamar Gov (Figure 1). The rhino evokes colonialism, as a (wild) creature that has long captured the imagination of the West. One may recall Albrecht Dürer’s famous 1515 woodcut print of a rhinoceros, which was created based on a textual description of the animal rather than direct observation (Sullivan, 2023). In Gov’s installation, the rhino also functions as a metaphor for the Western gaze and interest, whether in the context of big-game hunting, trophy collecting, or zoological and taxonomical scrutiny. The Romanic church in the installation represents the Western world and evokes the religious morals that were often invoked to justify colonial endeavours. The rhino, as massive as it is in Gov’s work, dominates the nave, occupying all available space—impossible to ignore and overwhelming to behold. Substituting the proverbial “elephant in the room,” the installation suggests that any church—or any other building symbolizing Western morals, norms, standards, or institutions—invites association with the rhino, reminding viewers of the co-constitution of modern postcoloniality. In this sense, the term “postcolonial nationality” itself may be read as paradoxical.

### 5.3 Nationality paradoxes in actions

As practical examples of the lived paradoxes of postcolonial nationality, I would like to briefly sketch three cases: Puerto Rico, Hong Kong, and Zimbabweans in South Africa.

Puerto Rican nationality displays several paradoxes (Duany, 2002). Puerto Ricans regard themselves as a nation, but they are citizens of the United States and, as such, are not politically sovereign. This creates a contradiction between political belonging to the US and cultural or national identification with Puerto Rico. Given its colonial past, Puerto Rico has Spanish, American, African, and Indigenous influences, which are themselves subject to complex hierarchizations that intersect national identity with ethnic and linguistic categories. Furthermore, Puerto Rican identity has a strong transnational or transterritorial dimension, as about half of the population resides on the US mainland. At the same time, Puerto Ricans living on the island do not enjoy the same political rights—particularly voting rights—as citizens living in the mainland United States. Especially in the US, experiences of being othered or racialized have led many Puerto Ricans to develop something akin to a Du Boisian “double consciousness”: they maintain a strong self-understanding as Puerto Rican (or “Boricua”), while at the same time preferring to retain US citizenship and the rights and entitlements that come with it (Laó-Montes, 2008).

Similarly, Hong Kong, which was a British colony in the past, now belongs politically to the People’s Republic of China while retaining a degree of autonomy as a Special Administrative Region. Hong Kong’s identity is paradoxical because many people reject both colonial and mainland political identities. While they often identify culturally with traditional Chinese heritage, they distance themselves politically from the Chinese state and instead develop a local Hongkonger identity within a Chinese sovereign framework (Tang et al., 2022). Many residents therefore identify primarily as Hongkongers rather than Chinese, creating tensions between state sovereignty and local identity, while ongoing political pressure from China has led to a growing diaspora and the perception of Hong Kong as a stateless nation (Iu, 2025).

A bit more detail will be provided on a study of my own (Kögel, 2024) on Zimbabwean migrants in South Africa, in which nationality emerged as a central and recurring theme. In the context of widespread hostility toward foreign nationals in South Africa—manifesting as structural discrimination, stigmatization, and fear—these migrants did not hide their Zimbabweanness. Rather, Zimbabweans describe themselves as educated, hardworking, humble, and morally upright—a strategy that functions as both a source of self-esteem and a defensive tool. Beyond these psychological effects, their nationality also carries practical significance: Zimbabwean citizenship provides the only formal claim to rights, opportunities, and belonging in contexts such as employment, education, or access to loans. Even when nationality produces exclusion, it simultaneously grants a legitimate position in political and social discourse.

Thus, Zimbabwean migrants in South Africa use their nationality strategically—as a retreat, a marker of positive identity, a justification for claims, and a standpoint from which rights and belonging can be asserted. The term “Zimbabwean” functions both as a label and a form of identification, whose content shifts depending on the enunciator, yet consistently enables migrants to assert equality, entitlement, and legitimacy within a hostile environment.

Exclusion from rights, resources and opportunities in South Africa may be enacted either on the basis of nativity or on that of residence and belonging, with one criterion often invoked when the other proves insufficient. Such dynamics produce paradoxical enactments of nationality—for instance when individuals emphasize their national identity even while facing discrimination on that very basis. Under such circumstances, nationality may become the only available strategic resource through which legitimate political claims can be articulated within a discourse that equates belonging with formal citizenship. As a result, a fundamentally humane agenda is reframed as a national cause, bringing self-identification and external categorization into disruptive alignment.

## 6 Conclusion

In order to make sense of the functioning of nationality—i.e. its performance as national identity (including differentiation between nationalities) and its constitutive distribution of belonging—it has been described as a floating signifier. As such, it precludes any essential meaning. Consequently, nationality is not simply contingent but arbitrary by necessity. What we commonly perceive and experience are particular nationalities based on mutual exclusion and differentiation; these may therefore appear essential, or be made to appear so, regardless of whether they are held to be contingent.

Being a floating signifier is not something that can be directly observed as such; rather, it presupposes its analysis as a (historical) process (schismogenesis). Understood in this way, it allows us to assess its effects and immediacy.^8^ The fact that these two dimensions operate according to their own logics results in various constellations and paradoxical effects and realities.

In postcolonial contexts, the legacies of colonial structures amplify these contradictions, making the tensions between belonging, recognition, and identity particularly pronounced. *Post*colonial nationality—beyond its temporal dimension—is not a form of nationality that constitutes a categorically different phenomenon. Yet, the postcolonial experience highlights the universal instability of the concept while revealing how historical and structural factors shape its lived realities.

## Data Availability

The original contributions presented in the study are included in the article/supplementary material, further inquiries can be directed to the corresponding author.

## Footnotes

1 By this, I mean that nationality is primarily regarded as a quality of the nation-state rather than of the nation. At the same time, the nation has been described as an idea adopted and incorporated by states for political and strategic reasons (Hobsbawm, 1992; Anderson, 2006).

2 Internally divided by class (but also, for example, by race or ethnicity), the nation functions ideologically to mask these divisions by presenting the population as a unified “people” (Balibar, 1990).

3 A detailed account for this argument can be found in Kögel, (2025).

4 Stuart Hall (1996a, p. 613) emphasizes that part of the “narrative of the nation”, which encompasses continuity, traditions and historicity, is the “foundational myth.” This myth illustrates “a story which locates the origin of the nation, the people and their national character so early that they are lost in the mists” (Hall, 1996a, p. 614). Since the supposed origin is so vague, it is easy to relate it to any suitable narrative. Given all these elements that are worked into the nation, its tradition, history, myths, etc. the nation appears not only as an “imagined,” but also an “interpretive community” (Said, 1983; Joseph, 2004).

5 Žižek uses the term “reflexive” signifier instead of “floating” signifier.

6 That identity is not essential but fragile, fragmented, fluid, etc., has been argued by a range of scholars and has rather become the mainstream understanding of identity by now, a view aptly problematized by Brubaker and Cooper (2000).

7 Georges Labica (2025) emphasizes Fanon’s positive understanding of the “national,” expressed in concepts such as “national consciousness,” “national struggle,” and “national culture,” in contrast to his cautious assessment of nationalism, which he particularly rejects when understood as a mere “defense of heritage.” Frantz Fanon writes in The Wretched of the Earth that “National consciousness, which is not nationalism, is the only thing that will give us an international dimension” (1963, p. 247). For Fanon, each national culture must fight for its freedom in pursuit of universal values that, upon independence, give way to humanism and international solidarity.

8 Nationality, as outlined above, provides little help in better understanding the concept of the nation. It carries the dimensions of identity and belonging, whether it refers to the nation or the nation-state; it conveys belonging regardless of whether this is formalized as citizenship. As such, it does little to further elaborate these notions or their differentiation. However, the social logic outlined for nationality (such as differentiation and distribution) may be applied to the notion of the nation and may point towards certain explanations.
